# Tick abundance, pathogen prevalence, and disease incidence in two contrasting regions at the northern distribution range of Europe

**DOI:** 10.1186/s13071-018-2890-9

**Published:** 2018-05-22

**Authors:** Atle Mysterud, Vetle Malmer Stigum, Ingrid Vikingsdal Seland, Anders Herland, W. Ryan Easterday, Solveig Jore, Olav Østerås, Hildegunn Viljugrein

**Affiliations:** 1 Centre for Ecological and Evolutionary Synthesis (CEES), Department of Biosciences, University of Oslo, P.O. Box 1066 Blindern, NO-0316 Oslo, Norway; 2 Department of Food, Water, Zoonotic & Vector-borne Infections, The Norwegian Public Health Institute, P.O. Box 4404 Nydalen, NO-0403 Oslo, Norway; 3 Department of the Norwegian Cattle Health Services, TINE Norwegian Dairies BA, NO-1431 Ås, Norway; 4 Norwegian Veterinary Institute, P.O. Box 750 Sentrum, NO-0106 Oslo, Norway

**Keywords:** Anaplasmosis, Babesiosis, *Ixodes ricinus*, Lyme borreliosis, Pathogen prevalence, Tick questing density

## Abstract

**Background:**

Emergence of tick-borne diseases is impacting humans and livestock across the Northern Hemisphere. There are, however, large regional variations in number of cases of tick-borne diseases. Some areas have surprisingly few cases of disease compared to other regions. The aim here is to provide a first step towards a better understanding of such contrasting regional patterns of disease emergences at the northern distribution range of *Ixodes ricinus* in Europe.

**Methods:**

We compare disease incidence, vector abundance and pathogen prevalence in eastern and western Norway differing in the number of tick-borne disease cases. First, we analysed the incidence of Lyme borreliosis in humans, tick-borne fever (anaplasmosis) in sheep and anaplasmosis and babesiosis in cattle to verify if incidence differed. Secondly, we analysed extensive field data on questing tick density, pathogen prevalence, as well as the broad spatial pattern of human and livestock distribution as it may relate to tick exposure.

**Results:**

The incidences of all diseases were lower in eastern, compared to western, Norway, but this was most marked for the livestock diseases. While the prevalence of *Borrelia burgdorferi* (*sensu lato*) in ticks was similar in the two regions, the prevalence of *Anaplasma phagocytophilum* was markedly lower in eastern, compared to western, Norway. We found overall a lower abundance of questing nymphs in the east. In the east, there were cases of babesiosis in cattle where anaplasmosis was absent, suggesting absence of the pathogen rather than differences in exposure to ticks as part of the explanation for the much lower incidence of anaplasmosis in eastern Norway.

**Conclusions:**

Many factors contribute to different disease incidence across ecosystems. We found that regional variation in tick-borne disease incidence may be partly linked to vector abundance and pathogen prevalence, but differently for human and livestock diseases. Further studies are needed to determine if there is also regional variation in specific genospecies and strain frequencies differing in pathogenicity.

**Electronic supplementary material:**

The online version of this article (10.1186/s13071-018-2890-9) contains supplementary material, which is available to authorized users.

## Background

Understanding the causes of emergence of vector-borne diseases over the last few decades is an area of intensive research [1, 2]. Lyme borreliosis is now the most common vector-borne disease in the northern hemisphere, with some 300,000 human cases annually in the USA and some 85,000 in Europe [3]. Other common tick-borne diseases in Europe are anaplasmosis (tick-borne fever) in sheep and cattle caused by the bacterium *Anaplasma phagocytophilum* [4–6] and babesiosis in cattle caused by the protozoan *Babesia divergens* [7, 8]. Lyme borreliosis is the most well-studied of the tick-borne diseases both in North America and Europe [9, 10]. Variation in disease incidence may come from variation in disease hazard, the (nymphal) vector abundance multiplied by the pathogen prevalence, as well as the level of exposure to ticks and the pathogen transfer process [2, 11]. These broad factors are in turn affected by a number of underlying factors such as abundance and competence of specific vertebrate transmission hosts for pathogenic genospecies or strains as well as their competence for the vector. We know little about which of these factors causes variation in the regional emergence of other tick-borne diseases, particularly in livestock.

The pathogens causing Lyme borreliosis, anaplasmosis and babesiosis are vectored by the same generalist ticks from the genus *Ixodes*. The species involved is *I. ricinus* in Europe, west Asia and north Africa, *I. persulcatus* in Asia and eastern Europe, and *I. scapularis* and *I. pacificus* in North America [12]. Most research has naturally focused on understanding the ecology and epidemiology in areas with high incidence of disease. The pathogens causing Lyme borreliosis, *Borrelia burgdorferi* (*sensu lato*) complex, are hosted by vertebrates that are habitat generalist and hence being ubiquitous [13, 14]. Therefore, low incidence of Lyme borreliosis are usually linked to low abundances of the vector [15]. Only a few studies have compared areas of high and low incidence of tick-borne diseases, but this yielded an important understanding of how tick life history affected Lyme borreliosis dynamics [15, 16]. For livestock diseases, the distribution of cases appears more spatially variable [17]. For anaplasmosis and babesiosis, low disease incidence can also stem from a lack of transmission hosts for specific pathogenic strains in a given region [11]; therefore pathogen distribution is limiting the disease. For example, it is suggested that the pathogenic strain of *A. phagocytophilum* causing anaplasmosis in livestock are linked to transmission in red deer and not roe deer [17]; hence anaplasmosis is predicted in regions with red deer [11]. Regional differences in disease incidence may also be driven by variation in exposure to ticks.

**Fig. 1:**
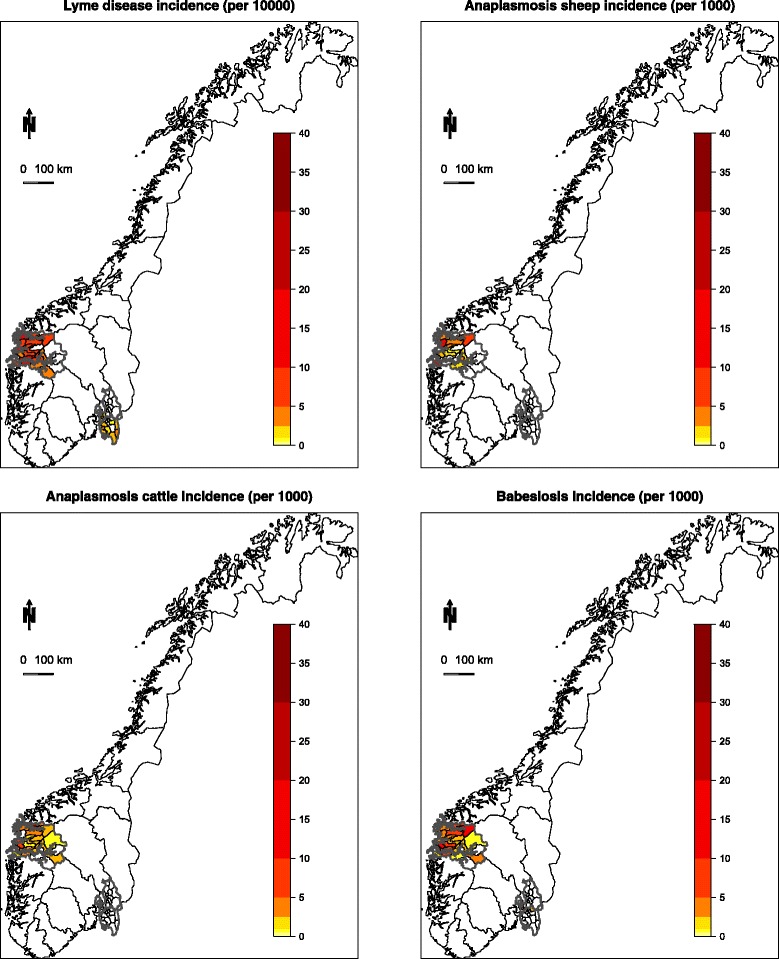
The incidence of Lyme borreliosis in humans, anaplasmosis in sheep and anaplasmosis and babesiosis in cattle in the western and eastern region of Norway

Tick-borne diseases are emerging across the northern distribution ranges of *I. ricinus* in Europe [18–20]. In Norway, Lyme borreliosis, anaplasmosis and babesiosis are all emerging [21], but there are contrasting spatial patterns across diseases [11, 20]. The general emergence has been linked to increased spatial distribution of the vector at these northern latitudes [21], which in turn can be linked to both climate change and dense deer populations [20]. The regional variation in tick-borne disease cases is less well understood. The incidences of tick-borne diseases are high along the west coast, while areas along the eastern part of Norway have rather few cases of all the tick-borne diseases (Fig. 1). In this study, we aim to gain insight into regional variation in limitation of tick-borne diseases by contrasting the eastern part to the western part of Norway. We analyse data on questing *I. ricinus* tick abundance, the prevalence of the pathogens *B. burgdorferi* (*s.l.*) and *A. phagocytophilum* along transects in our two regional study sites, and analyse statistics of incidence of Lyme borreliosis in humans, anaplasmosis in sheep and both anaplasmosis and babesiosis in cattle covering a broader area at the scale of municipality. We aim to explore if fewer cases in one region (eastern Norway) compared to another (western Norway) is due to lower abundance of questing ticks, prevalence of pathogens causing the diseases, or if broad spatial distribution of humans and livestock are likely to make them differently exposed to ticks. We suggest insight can be gained by using a comparative approach with several tick-borne diseases sharing the same vector, but differing in terms of pathogens, transmission hosts and pattern of exposure [11].

## Methods

### Study areas

The eastern study area is located in the southeast of Norway, in Akershus and Østfold county (Fig. 1). The landscape morphology and topology in the region are characterized by low variation in elevation ranging from 20 to 221 m above sea level (masl). The average annual temperature is 3.4 °C and average total precipitation is 735 mm (Norwegian meteorological station no.03780). The area is mostly situated in the boreonemoral zone, and is a transitional zone between the nemoral deciduous forest areas and the boreal coniferous areas [22]. The study area consists mainly of mixed coniferous stands with either Scots pine (*Pinus sylvestris*) or Norway spruce (*Picea abies*) as dominant tree species. Deciduous trees such as birch (*Betula* spp.) and alder (*Alnus incana*) are common. Forests are heavily managed for forestry purposes. Roe deer and moose are common in the area.

The western study area is in Sogn & Fjordane county, western Norway. The climate in this area consists of cool summers and mild winters with an average annual temperature of 6 °C and yearly precipitation of 2270 mm (Norwegian meteorological station no 57170). The area is situated within the boreonemoral zone [22], but dominated by deciduous trees mainly birch (*Betula* spp.) at higher elevation and alder (*Alnus incana*) at lower elevation. There are scattered Norway spruce forests planted for forestry. Red deer are common in the area.

### Study design and flagging for ticks

Eastern study site (with few disease cases): The study design in Akershus/Østfold was guided by prior knowledge of assumed tick distribution. We aimed to have a variable distance to the coast (0.2–47.7 km), distance to inland lake (> 0.5 km^2^) (0–17.7 km) and elevation (20–221 masl). We flagged all 58 transects in 2014, while this was reduced to 37 in 2015 and 28 in 2016 due to logistical constraints. In comparisons of tick abundances and pathogen prevalences across years, we restricted to the main distribution of ticks and the 28 transects taken all years. Each transect consists of 9 plots in a triangle to make sampling efficient [23].

Western study site (with many disease cases): Part of the data from the Sogn & Fjordane county in Norway has been presented elsewhere [20, 24–26]. We here add 5 years of data on prevalence of *A. phagocytophilum* and 2 years for *B. burgdorferi* (*s.l.*) in questing ticks relative to previous work. The same 34 transects spanning variation in distance to coast (0.1–25 km) and elevation (25–482 masl) has been flagged yearly in the period 2009–2016. The transects in this area consist of 12 plots along a straight line within home range of GPS-marked red deer [25].

We sampled for questing ticks in spring or early summer in both areas. This was done during May in Sogn & Fjordane and mainly June in Akershus (76% of data from June, remaining data from July and early August in 2014). In both areas, we followed our previously described procedure of sampling questing ticks by aid of the cloth-lure or flagging method [27]. We swept a white cotton cloth (1 × 0.5 m) attached to a strip of wood, slowly through the vegetation. This was done for a 2 m wide area four times (hence covering about 4 m^2^) before the cloth was examined for ticks and repeated five times in each plot, thus covers an area of approximately 20 m^2^. We counted adult (male/female) and nymphal ticks attaching to the cloth. The cloth was replaced when it became wet or dirty. All the ticks were removed with a tweezers and put in a tube with ethanol and labeled with location number and date. After field work, ticks were dried and stored in tubes with silica beads at -20 °C to avoid degradation of DNA used for screening of pathogens.

### Pathogen determination - the PCR protocol

The presence of the tick-borne pathogens *B. burgdorferi* (*s.l.*) and *A. phagocytophilum* was determined using an established protocol at our lab at the Department of Biosciences, University of Oslo [20, 24]. The DNA extraction method is based on Allender et al. [28] and optimized for use on ticks. Detection of *A. phagocytophilum* and *B. burgdorferi* (*s.l.*) in ticks was conducted by using a multiplex real-time PCR assay on a Roche Light Cycler 96 Real-Time PCR instrument [29].

### Disease incidence

We use here the same data on incidence of Lyme borreliosis in humans, anaplasmosis and babesiosis in cattle and anaplasmosis in sheep as in our previous analysis at large scales across Norway [11, 20]. We here briefly describe the data; a more detailed description and discussion of the data in terms of determining the disease, treatment and reporting as potential biases is found in the appendix of Mysterud et al. [11]. To enhance comparisons, we removed the city of Oslo and the part of Akershus county being suburbs west of Oslo (Bærum and Asker municipality), as these are densely populated areas and we have no surveys of ticks in those regions.

#### Lyme borreliosis in humans

Human cases of tick-borne Lyme borreliosis were retrieved from the Norwegian Surveillance System for Communicable Diseases (MSIS) for the time period 2006 to 2015. Laboratory-confirmed (disseminated; stage 2–3) Lyme borreliosis is a notifiable disease and reported to the Norwegian Institute of Public Health (NIPH) [21].

#### Anaplasmosis and babesiosis in cattle

Records of the tick-borne diseases bovine babesiosis and anaplasmosis are available from the Norwegian Cattle Health Recording system (NCHRS) for the time period 2006 to 2015 [11, 30]. The records include only animals that were treated by veterinarians and this system is considered to be highly reliable in terms of reporting rates and quality of the system [30]. The registration is based on clinical symptoms rather than confirmed cases based on laboratory diagnostics. Clinical symptoms are less clear for anaplasmosis than babesiosis. Nevertheless, at the scale of the whole of Norway, incidences of anaplasmosis and babesiosis in cattle were spatially correlated [11], and we here only consider spatial contrasts less likely to be affected by these difficulties. Incidences were obtained by adjusting for number of outfield-grazing cattle.

#### Anaplasmosis in sheep

Cases of the tick-borne disease anaplasmosis were retrieved from the Sheep Recording System database (“Sauekontrollen”) in Norway for the time period 2006 to 2015. This database includes only records in which the animals were treated by veterinarians and reported by the sheep farmer. Due to this difference in way of reporting and the lower economic value of sheep compared to cattle, there is likely more underreporting of disease in this database compared to for cattle, in addition to that clinical symptoms of anaplasmosis is less clear. However, this is unlikely to affect the spatial contrasts we analyse here. As in a previous analysis, we included a covariate (“health recordings”; number of all diseases being reported) to control for potential bias of reporting between municipalities [11]. Incidences were obtained by adjusting for number of ewes registered in the database.

### Other spatial and temporal covariates

Population densities are calculated relative to total land area for sheep, cattle and humans (Additional file 1: Figure S1). Densities of cervids are the harvest number divided by the qualifying habitat used in deer management for roe deer, red deer and moose (Additional file 1: Figure S2), as is a much used index of population density of cervids, see for further details [20].

Tick density is strongly related to distance from the coast and elevation in Norway [21, 25, 31]. We therefore retrieved data on distance from the fjord for each municipality [20]. Ticks are abundant up to approximately 200–250 masl in Norway; therefore, we produced metrics on the proportion of area below 200 m for each municipality. In addition, we retrieved data on the proportions of forested area, agricultural land and human settlement in each municipality from Statistics Norway.

### Statistical analysis

The statistical analysis was performed in R version 3.4.1 [32]. To assess whether the overall regional differences were significant, we ran simple comparisons only including region as a categorical variable (reported in Table 1). The data on pathogen prevalence was analysed using logistic regression at the scale of transects. The data on tick counts were analysed with generalized mixed effects models (GLMMs) with negative binomial distribution using the package ‘glmmADMB’ [33]. For analysis of tick counts, we used ‘transect ID’ as a random term, as the response variable was abundance of nymphs for each plot of 20 m^2^. As covariates and their scale ranges differed for the two areas, we performed separate analyses when including spatial covariates. In the east region model, we used data from 2014 encompassing all transects, and included season (June *vs* July/August), the distance to nearest lake, and the distance to the coast, which is a main proxy for climate in these coastal areas. We did not add elevation to this model, as elevation was correlated (r = 0.590) with distance to the coast in the east. In the west, the full model included annual variation (2009–2016), elevation and the distance to the coast. All data was from the same month (May) in the west region.

**Table 1:** A summary of patterns and hypotheses to explain the regional variation of tick-borne disease cases in eastern and western Norway. All numbers are means for the entire data period (see other tables for detail). Densities are relative to total land area (in km^2^ for 2012)

Patterns/mechanisms	Eastern Norway	Pattern/conclusion	Western Norway	*Z* or *t*	*P*
Cases (sum 2006–2015)					
Lyme disease	96	<	133	2.09	0.041
Anaplasmosis sheep	0	<	194		
Anaplasmosis cattle	0	<	134		
Babesiosis cattle	3	<	214	2.97	0.004
Population sizes					
Humans	648,071	>	107,542	-4.50	< 0.001
Sheep (registered ewes)	4324	<	24,728	6.05	< 0.001
Cattle (outfield grazing)	4963	<	22,697	6.91	< 0.001
Population densities (mean)					
Humans	74.76	>	5.73	4.31	< 0.001
Sheep	0.49	<	1.32	-3.30	0.002
Cattle	0.56	<	1.21	-2.88	0.006
Incidence (mean)					
Lyme disease (per 100,000)	1.49	<	12.41	6.12	< 0.001
Anaplasmosis sheep (per 10,000)	0	<	7.90		
Anaplasmosis cattle (per 10,000)	0	<	5.83		
Babesiosis cattle (per 10,000)	0.72	<	9.32	4.38	< 0.001
Nymphal tick abundance (per 20 m^2^)	1.36	<	5.78	2.08	0.038
Pathogen prevalence in nymphs					
*B. burgdorferi* (*s.l.*)	11.4%	≥	11.3%	-1.364	0.172
*A. phagocytophilum*	1.0%	<	4.5%	-5.346	< 0.001

Analysis of disease incidences over the ten years (2006–2015) was performed by generalized linear models (GLMs) with negative binomial distribution. There were an insufficient amount of cases to do a spatial analysis of the livestock diseases in the eastern part of Norway. Variation in number of disease cases between regions may also arise if human or livestock numbers have a different spatial distribution, so that it impacts exposure to ticks. The spatial pattern of the population numbers and densities of humans, sheep and cattle was analysed with GLM with quasi-poisson distribution (log link) and gamma distribution (inverse link), respectively. Note that the inverse link of the gamma distribution for population densities causes t-values to have a reversed sign. The Akaike Information Criterion (AIC) was used in model selection to select the most parsimonious model. Collinearity was assessed by calculating variance inflation factors (VIF’s) [34], and only variables having VIFs < 4 were retained in a given model. Model fit was evaluated by plotting the residuals against the predicted values and by plotting the residuals of the final models of incidence against each of the explanatory variables. Explanatory variables were natural log-transformed or square root-transformed to linearize their relationships with the response variable when needed to improve fit.

## Results

### Questing tick abundance

There was a markedly higher number of questing nymphs in western compared to eastern Norway all three years, while the difference was not as high for adult ticks (Table 2). The mean abundance of nymphs in the east averaged 1.36 per 20 m^2^ for all years, with the maximum of 6 nymphs per 20 m^2^. Abundance was 0.58, 1.86 and 1.63 nymphs per 20 m^2^ for years 2014, 2015 and 2016, respectively. The abundance of ticks in the west averaged 5.78 nymphs per 20 m^2^ if only considering plots below 200 masl in order to compare a similar elevation range, ranging in mean from 2.9 to 8.3 nymphs per 20 m^2^ across years 2009–2016 and with a maximum of 191 nymphs per 20 m^2^.

**Table 2:** The abundance of *Ixodes ricinus* ticks per 20 m^2^ from flagging in two regions differing in incidence of tick-borne diseases; ‘east’ (Akershus and Østfold) and ‘west’ (Sogn & Fjordane) in Norway. We report full samples sizes, while only transects flagged all years were considered and restricted to below 200 masl to ease comparison of densities across areas

Region	Year	Nymphs			Adult males			Adult females		
		*n* ^b^	Mean^a^	SD^a^	*n* ^b^	Mean^a^	SD^a^	*n* ^b^	Mean^a^	SD^a^
‘East’ (Akershus and Østfold)	2014	199	0.58	4.26	19	0.04	0.22	14	0.02	0.13
	2015	577	1.86	1.06	58	0.21	0.66	49	0.18	0.62
	2016	412	1.63	2.63	54	0.21	0.60	42	0.17	0.48
	All	1188	1.36	3.00	131	0.16	0.54	105	0.12	0.46
‘West’ (Sogn & Fjordane)	2009	1961	8.29	15.12	101	0.42	1.06	99	0.40	0.90
	2010	1925	8.08	16.31	83	0.33	0.64	84	0.33	0.73
	2011	1408	5.95	13.23	57	0.27	1.93	33	0.15	0.50
	2012	1701	6.80	11.12	49	0.19	0.53	53	0.21	0.63
	2013	666	2.87	6.78	24	0.12	0.43	26	0.12	0.46
	2014	750	3.20	6.05	33	0.14	0.41	24	0.09	0.31
	2015	1195	5.54	15.33	32	0.14	0.55	40	0.17	0.80
	2016	1313	5.39	9.73	53	0.25	0.86	43	0.19	0.56
	All	10,919	5.78	12.41	432	0.23	0.94	402	0.21	0.64

^a^Mean and SD is at scale of plots (20 m^2^)

^b^*n* is total number collected for all transects (28 in ‘east’, 34 in ‘west’)

In the eastern part of Norway, the nymphal tick abundance decreased as distance to the coast increased (*Z* = -3.42, *P* < 0.001), and abundance was higher in June than in July and August pooled (*Z* = -5.45, *P* < 0.001). Including distance to large lake to the model improved the AIC marginally (Additional file 1: Table S1), but was not significant when added to the model (*Z* = -1.43, *P* = 0.153). In western Norway, the nymphal tick abundance similarly decreased as distance to the coast (*Z* = -3.00, *P* < 0.001) and elevation (*Z* = -2.77, *P* < 0.001) increased, and there was marked interannual variation (*P* < 0.001). The full model was markedly better than other models as judged by AIC (Additional file 1: Table S1).

### Pathogen prevalence

The prevalence of *B. burgdorferi* (*s.l.*) was similar in the two areas, at least for nymphs (*t* = -1.364, *P* = 0.172). Prevalence averaged 11.4% in nymphs (*n* = 872), 14.7% in adult males (*n* = 109) and 24.2% in adult females (*n* = 91) in the eastern part of Norway, while it was 11.3% in nymphs (*n* = 2822), 10.9% in adult males (*n* = 348) and 11.2% in adult females (*n* = 340) in the western part of Norway (Table 1; Additional file 1: Table S2).

The prevalence of *A. phagocytophilum* was markedly higher in the western compared to the eastern study area (Table 1). The prevalence of *A. phagocytophilum* averaged 1.0% in nymphs, 7.3% in adult males and 3.3% in adult females in the east, while it was 4.5% in nymphs, 13.8% in adult males and 13.5% in adult females in the west (same sample sizes as for *B. burgdorferi* (*s.l.*); Additional file 1: Table S2).

### Incidence of disease

In the eastern part of Norway, the mean incidence of Lyme borreliosis was 1.49 per 100,000 humans, while incidence was 12.41 for the western part for years 2006–2015 (Table 1; Additional file 1: Table S3). There were no recorded cases of anaplasmosis among the (on average each year) 4324 in the sheep registry and 4963 outfield grazing cattle in east, while incidence was 7.9 per 10,000 sheep and 5.83 per 10,000 outfield grazing cattle in west. Incidence of babesioses in cattle was 9.32 per 10,000 outfield grazing cattle in west and 0.72 in east.

There was a significantly higher incidence of Lyme borreliosis in western compared to eastern Norway (Table 3, model selection results in Additional file 1: Table S4). Incidences decreased with increasing distance to coast and increased proportion of human settlement. Incidence also decreased with proportion of high elevation terrain and more so in the west than in the east. Babesiosis in the west decreased with distance to the coast, while it increased with increasing deer density in the municipality. Anaplasmosis in cattle also decreased with increasing distance to the coast (*P* > 0.05), and increased with proportion of agricultural areas. Anaplasmosis in sheep decreased with increasing distance to coast and also depended on the density of outfield-grazing sheep.

**Table 3:** Parameter estimates and test statistics for negative binomial models of incidence of tick-borne diseases in Norway 2006–2015. For Lyme borreliosis, the data include both western and eastern parts of Norway. For the livestock diseases, the analyses only include western Norway due to few livestock-disease cases in the east. Continuous variables were scaled (mean = 0, SD = 1)

Parameter	Estimate	SE	*Z*	*P*
Lyme borreliosis				
Intercept	-10.86	0.85	-12.80	< 0.001
Distance to coast	-1.00	0.34	-2.99	0.003
Region (‘west’ *vs* ‘east’)	3.47	1.06	3.27	0.001
Prop. area > 200 masl	-2.15	0.76	-2.83	0.005
sqrt(prop. area human settlement)	-0.55	0.18	-2.97	0.003
Region (‘west’ *vs* ‘east’)*(Prop. area > 200 masl)	1.71	0.75	2.29	0.022
Babesiosis cattle				
Intercept	-5.09	0.15	-32.89	< 0.001
Distance to coast	-0.35	0.15	-2.29	0.022
log(spatial deer density+0.001)	0.64	0.19	3.37	0.001
Anaplasmosis cattle				
Intercept	-5.33	0.22	-24.59	< 0.001
Distance to coast	-0.29	0.21	-1.38	0.169
sqrt(prop. area agricultural fields)	0.64	0.24	2.65	0.008
Anaplasmosis sheep				
Intercept	-6.25	0.35	-18.02	< 0.001
Distance to coast	-1.93	0.38	-5.07	< 0.001
Health recordings	0.56	0.25	2.25	0.024
log(density of outfield grazing sheep)	1.58	0.40	3.98	< 0.001

The spatial distribution patterns of livestock and humans may affect exposure to ticks. In the eastern part of Norway, although the density of sheep grazing outfields (*t* = -2.16, *P* = 0.04), number of registered ewes (*t* = 4.13, *P* < 0.001) and cattle grazing outfields (*t* = 2.37, *P* = 0.02) all increased as distance to coast increases, this was not the case for the densities of ewes (*t* = -0.49, *P* = 0.63) and outfield grazing cattle (*t* = 0.38, *P* = 0.71). Number (*t* = -2.40, *P* = 0.02) as well as density (*t* = 2.86, *P* = 0.007) of inhabitants decreased as distance to coast increases. Increasing distance to coast correlates with higher elevation making it difficult to separate their effects. In western Norway, density (*t* = -1.91, *P* = 0.07) and number (*t* = 3.02, *P* = 0.006) of ewes increased as distance to coast increased. This was not significant for density of outfield-grazing sheep (*t* = -0.44, *P* = 0.66), but marginally significant for the number of sheep grazing outfields; *t* = 1.66, *P* = 0.11) and cattle grazing outfields (number: *t* = 1.59, *P* = 0.13, densities: *t* = -1.56, *P* = 0.13). Number (*t* = 0.26, *P* = 0.80) and densities (*t* = 0.90, *P* = 0.38) of inhabitants did not show a significant pattern with distance to coast.

## Discussion

Emergences of tick-borne diseases may be due to variation in a number of factors, such as abundance of ticks, prevalence of pathogens, level of pathogenicity, as well as the level of exposure to ticks through land use for livestock or livelihoods connected to forest or recreational activities in humans. We have contrasted two regions towards the northern distribution range of *I. ricinus* in Europe differing largely in number of cases of Lyme borreliosis in humans, anaplasmosis in sheep and anaplasmosis and babesiosis in cattle. We confirmed that incidence of all diseases, not just the number of cases, was lower in the eastern region compared to the western region of Norway. The most notable finding was the very low prevalence of *A. phagocytophilum* in questing nymphs in the eastern region, likely being at least part of the explanation for the low incidence of anaplasmosis in the region.

### The role of pathogen distribution patterns

Vector-borne diseases share the common feature of relying on the presence of the vector. However, the different pathogens differ largely in their biology and the extent to which they rely on single or many transmission hosts with or without a broad geographical distribution. The distribution of tick-borne diseases may therefore be more limited than the distribution of the vector. For example, the spatial distribution of tick-borne encephalitis virus (TBEV) in Europe is very limited, likely reflecting the need for specific conditions for the co-feeding transmission cycle. Transmission of TBEV requires co-feeding of at least 10 larvae and one infected nymph on the same host to have a continuing epidemiological cycle [35], and this is linked to quite specific temperature conditions [36]. In contrast, the presence of Lyme borreliosis can be quite well-predicted based on the presence of the vector. There is little doubt that climate warming directly affecting the life history of *I. ricinus* is a major factor influencing the general emergence of Lyme borreliosis in Europe [37–39]. Increasing incidence of Lyme borreliosis is now reported in the northern parts of Europe, both in Norway [20] and Finland [19]. If there is *I. ricinus* in an area, the pathogen *B. burgdorferi* (*s.l.*) causing Lyme borreliosis will usually be present, as the transmission hosts are widespread [40]. Consistent with this, the prevalence of *B. burgdorferi* (*s.l.*) in questing nymphs was similar in the two areas (11.3% in western and 11.4% in eastern Norway).

### The pattern of tick questing abundances

Disease hazard depends on both the tick questing abundances and their pathogen prevalence levels, often measured as number of infected nymphs [13]. Determining the abundances of ticks with the flagging method is difficult as such data also reflect patterns of tick activity, which is influenced by a combination of photoperiod and prevailing weather [41, 42]. Hence, tick activity varies across seasons, years and regions. We found overall higher abundance of questing nymphs in the west (5.78/20 m^2^), compared to east (1.36/20 m^2^), which will for a similar prevalence, as for *B. burgdorferi* (*s.l.*), yield a higher disease hazard in the west of Norway. This higher abundance of questing ticks in the west may contribute to the more than 8.3-fold higher Lyme borreliosis incidence in the west (12.4 per 100,000) compared to the east (1.49 per 100,000) of Norway. Our time series of field data were too short to link disease hazard to incidence over years, and we relied on spatial contrasts for inference. There was marked annual variation in tick questing abundances in both regions, so this may certainly contribute to explain annual variation in disease incidence.

We found almost twice as many adults relative to nymphs in the east compared to the west (Table 2). Though we cannot explain this pattern with our current knowledge, it may suggest that life-cycles and mortalities differ between regions. The west coast of Norway is warmer and more humid than the eastern area, which has a more inland climate. Temperature and not humidity was limiting for tick questing activity along the west coast [42], while no study was available from the whole season in our eastern study area. A limitation of our study was the lack of data on the full seasonal variation in questing activity, and that sampling periods also differed, being May in west and mainly June in east. Further, it is also possible that the distribution of specific genospecies of *B. burgdorferi* (*s.l.*) play a role, as they may cause different clinical symptoms [43]. Lastly, we cannot disregard a role of differences in human exposure to ticks between the regions [44]. The incidence of tick-borne encephalitis in eastern Europe was linked to activity in forests, which again was driven by socio-economic factors [45]. There was decreased incidence of Lyme borreliosis with more human settlement within a municipality in our study. Clearly, more detailed studies of how humans and livestock get exposed to ticks in eastern compared to western Norway would be fruitful.

### Spatially variable prevalence of *A. phagocytophilum*

Anaplasmosis is a major problem in many areas of Europe, as *A. phagocytophilum* is also regarded a widely-spread pathogen [6]. Along the west coast of Norway, anaplasmosis in sheep is a major problem [46]. Infection of *A. phagocytophilum* causes mortalities and reduces body growth in lambs [47]. Exposure to ticks is often linked to grazing infields in early spring [48], before sheep are sent for summer grazing at higher elevation with lower abundances of ticks [42]. There are often vague clinical symptoms with anaplasmosis and the disease may be underreported. Nevertheless, the incidence of anaplasmosis in sheep was high in the western region, with some 10 cases per 10,000 sheep, while there was no recorded case in the eastern region during the study period (2006–2015, Table 1, Fig. 1). In contrast to the even distribution of the pathogen *B. burgdorferi* (*s.l.*) causing Lyme borreliosis, the prevalence of *A. phagocytophilum* was markedly higher in the west, compared to the east, of Norway.

While the transmission hosts of *B. burgdorferi* (*s.l.*) is well-known [40, 49], this is still debated for *A. phagocytophilum* [17, 50]. Red deer is often inferred as the main transmission host of *A. phagocytophilum* in Norway [51], and the distribution of red deer is mainly associated with the west coast of Norway [52] (Additional file 1: Figure S2). Prevalence of *A. phagocytophilum* along the west coast was as high as 75% in red deer [53], and the prevalence of *A. phagocytophilum* in ticks is higher in areas with high abundance of deer in Norway [54, 55]. However, high levels of prevalence (76%) were also found in roe deer in France [56], and both roe deer and moose are abundant hosts in the eastern parts of Norway (Additional file 1: Figure S2). Consistent with this, most roe deer surveyed had *A. phagocytophilum* infection in the eastern region (A. Mysterud et al., unpubl. data). It is therefore surprising that we see such low prevalence in nymphs. We hypothesize that larval host selection may play a role in pathogen prevalence levels in nymphs. Tick larvae are quite abundant on red deer on the west coast [57], while we found no larvae on roe deer in the eastern area (A. Mysterud et al., unpubl. data). Samples from roe deer were from early spring, so it is uncertain if this only reflects late onset of larval questing or differences in host selection. Further studies are therefore required to understand if this can provide, at least part of, a possible explanation for the lower proportion of *A. phagocytophilum* in nymphs and the resulting lower incidence of anaplasmosis in eastern compared to western Norway. If larvae feed mainly on other vertebrates with lower/no *A. phagocytophilum* infection, there will be few infected nymphs, assuming co-feeding is not a main route of transmission [36]. However, if nymphs are feeding on roe deer, then adult ticks may have higher prevalence of *A. phagocytophilum*, and to some extent this was the pattern we found (Table 1).

Livestock often receive many more tick bites than a human, and even a low prevalence may pose a high risk of infection. For *A. phagocytophilum*, strains differ in their pathogenicity to cattle [58]. It has been suggested four main circulating strains of *A. phagocytophilum* in Europe with reservoirs in: (i) red deer and livestock; (ii) roe deer; (iii) rodents; and (iv) birds [17, 50]. Since red deer share the same strain as livestock, and since red deer are largely absent from the eastern study area (Additional file 1: Figure S2), this may provide an additional explanation of the absence of anaplasmosis in this area. However, both the number of *A. phagocytophilum* strains as well as the pathogenicity of the strains for livestock in Norway is still regarded as uncertain [51].

## Conclusions

We found that regional variation in number of disease cases was linked to differences in several factors. A shared factor for the lower incidence of all diseases in the east of Norway was lower abundances of questing tick nymphs. For livestock diseases, but not for Lyme borreliosis, the fewer cases in the east compared to the west is also partly due to much lower populations of sheep and cattle. Differences in exposure to ticks cannot explain the absence of anaplasmosis in cattle in the eastern part of Norway, as there were cases of babesiosis, even though they were few in number. The low prevalence of *A. phagocytophilum* found in questing ticks in the east may contribute to lower diseases incidence. Our study has provided a first step towards explaining some of the regional differences in disease incidence seen in the northern part of Europe.

## Additional files

Additional file 1: **Table S1.** The mean abundance of *Ixodes ricinus* ticks per 20 m^2^ from flagging in two regions differing in incidence of tick-borne diseases; ‘east’ (Akershus and Østfold) and ‘west’ (Sogn & Fjordane) in Norway. Only transects flagged all years were considered and restricted to below 200 masl to ease comparison across areas. **Table S2.** The prevalence of the tick-borne pathogens *B. burgdorferi* (*s.l.*) and *A. phagocytophilum* from ‘east’ (Akershus and Østfold) and ‘west’ (Sogn & Fjordane) of Norway. **Table S3.** The number of cases and incidence (inc) of three tick-borne diseases, Lyme borreliosis, anaplasmosis in sheep, anaplasmosis and babesiosis in cattle, from areas ‘west’ (Sogn & Fjordane) and ‘east’ (Akershus and Østfold) in Norway. Incidence reported per 100,000 inhabitants, per 10,000 registered ewes and per 10,000 cattle. CattleOut denote those grazing on the outfields, and incOut is the incidence restricted to those cattle grazing outfield. **Table S4.** Results from model selection with AIC for the analysis of variation in incidence of tick-borne diseases with negative binomial mixed models for east and west of Norway. **a** Lyme borreliosis; **b** Babesiosis in cattle; **c** Anaplasmosis in cattle; **d** Anaplasmosis in sheep. Municipality ID was included as a random term to account for sampling design. **Figure S1.** The population density of humans, sheep and cattle in the western and eastern region of Norway. **Figure S2.** The population density of red deer, moose and roe deer in the western and eastern region of Norway. (DOCX 653 kb)

## Abbreviations

*B. burgdorferi* (*s.l.*): *Borrelia burgdorferi* (*sensu lato*)
masl: meters above sea level
