# Supplementary material for: Tick abundance, pathogen prevalence, and disease incidence in two contrasting regions at the northern distribution range of Europe
Source: Parasit Vectors. 2018 May 22;11:309. doi: 10.1186/s13071-018-2890-9 (PMC5964723; doi:10.1186/s13071-018-2890-9)
Supplement: Supplementary file 1 — Table S1. The mean abundance of Ixodes ricinus ticks per 20 m2 from flagging in two regions differing in incidence of tick-borne diseases; ‘east’ (Akershus and Østfold) and ‘west’ (Sogn & Fjordane) in Norway. Only transects flagged all years were considered and restricted to below 200 masl to ease comparison across areas. Table S2. The prevalence of the tick-borne pathogens B. burgdorferi (s.l.) and A. phagocytophilum from ‘east’ (Akershus and Østfold) and ‘west’ (Sogn & Fjordane) of Norway. Table S3. The number of cases and incidence (inc) of three tick-borne diseases, Lyme borreliosis, anaplasmosis in sheep, anaplasmosis and babesiosis in cattle, from areas ‘west’ (Sogn & Fjordane) and ‘east’ (Akershus and Østfold) in Norway. Incidence reported per 100,000 inhabitants, per 10,000 registered ewes and per 10,000 cattle. CattleOut denote those grazing on the outfields, and incOut is the incidence restricted to those cattle grazing outfield. Table S4. Results from model selection with AIC for the analysis of variation in incidence of tick-borne diseases with negative binomial mixed models for east and west of Norway. a Lyme borreliosis; b Babesiosis in cattle; c Anaplasmosis in cattle; d Anaplasmosis in sheep. Municipality ID was included as a random term to account for sampling design. Figure S1. The population density of humans, sheep and cattle in the western and eastern region of Norway. Figure S2. The population density of red deer, moose and roe deer in the western and eastern region of Norway. (DOCX 653 kb) [file 13071_2018_2890_MOESM1_ESM.docx]

**Additional file 1**

**Additional file 1: Table S1.** Results from model selection with AIC for the analysis of variation in tick abundance with negative binomial mixed models in (A) the east region for year 2014 and (B) the west region of Norway. x denotes if a variable was included. Transect ID was always included as a random term to account for sampling design with plots within transects.

| A. ‘East’ |  |  |  |  |
| --- | --- | --- | --- | --- |
| Season (June vs. July/Aug) | Distance to coast | Distance to lake | AIC | ΔAIC |
|  |  |  | 781.84 | 33.09 |
| **x** | **x** | **x** | 748.75 | 0 |
| **x** | **x** |  | 748.77 | 0.03 |
| **x** |  |  | 777.29 | 28.54 |
|  | **x** | **x** | 755.10 | 6.35 |
|  | **x** |  | 757.31 | 8.56 |
| B. ‘West’ |  |  |  |  |
| Year (2009-16 as categories) | Distance to coast | Elevation | AIC | ΔAIC |
| **x** | **x** | **x** | 18250.10 | 0 |
|  | **x** | **x** | 18519.54 | 269.44 |
| **x** |  | **x** | 18255.70 | 5.60 |
| **x** | **x** |  | 18255.30 | 5.20 |
| **x** |  |  | 18279.94 | 29.84 |
|  | **x** |  | 18524.82 | 274.72 |
|  |  | **x** | 18525.10 | 275.00 |

**Additional file 1: Table S2.** The prevalence of the tick-borne pathogens *B. burgdorferi* s.l. and *A. phagocytophilum* from ‘east’ (Akershus and Østfold) and ‘west’ (Sogn & Fjordane) of Norway. Sample sizes are slightly lower than for total number of flagged ticks in table 1.

| Area  pathogen | Year | Nymphs | | | Adult males | | | Adult females | | |
| --- | --- | --- | --- | --- | --- | --- | --- | --- | --- | --- |
|  |  | n_pos_ | n_tot_ | % | n_pos_ | n_tot_ | % | n_pos_ | n_tot_ | % |
| ‘East’ |  |  |  |  |  |  |  |  |  |  |
| *B. burgdorferi* s.l. | 2014 | 20 | 178 | 11.2 | 2 | 14 | 14.3 | 1 | 7 | 14.3 |
|  | 2015 | 61 | 453 | 13.5 | 9 | 52 | 17.3 | 8 | 47 | 17.0 |
|  | 2016 | 18 | 241 | 7.5 | 5 | 43 | 11.6 | 13 | 37 | 35.1 |
|  | sum | 99 | 872 | 11.4 | 16 | 109 | 14.7 | 22 | 91 | 24.2 |
| *A. phagocytophilum* | 2014 | 1 | 178 | 0.6 | 0 | 14 | 0.0 | 0 | 7 | 0.0 |
|  | 2015 | 3 | 453 | 0.7 | 2 | 52 | 3.8 | 1 | 47 | 2.1 |
|  | 2016 | 5 | 241 | 2.1 | 6 | 43 | 14.0 | 2 | 37 | 5.4 |
|  | sum | 9 | 872 | 1.0 | 8 | 109 | 7.3 | 3 | 91 | 3.3 |
| ‘West’ |  |  |  |  |  |  |  |  |  |  |
| *B. burgdorferi* s.l. | 2009^*^ | 11 | 125 | 8.8 | 4 | 54 | 7.4 | 1 | 60 | 1.7 |
|  | 2010^*^ | 65 | 386 | 16.8 | 13 | 80 | 16.3 | 15 | 79 | 19.0 |
|  | 2011^*^ | 84 | 668 | 12.6 | 3 | 29 | 10.3 | 5 | 24 | 20.8 |
|  | 2012^*^ | 48 | 417 | 11.5 | 11 | 51 | 21.6 | 10 | 55 | 18.2 |
|  | 2013 | 15 | 201 | 7.5 | 1 | 25 | 4.0 | 3 | 21 | 14.3 |
|  | 2014 | 37 | 289 | 12.8 | 4 | 33 | 12.1 | 0 | 22 | 0.0 |
|  | 2015^*^ | 41 | 355 | 11.5 | 0 | 31 | 0.0 | 2 | 39 | 5.1 |
|  | 2016 | 18 | 381 | 4.7 | 2 | 45 | 4.4 | 2 | 40 | 5.0 |
|  | sum | 319 | 2822 | 11.3 | 38 | 348 | 10.9 | 38 | 340 | 11.2 |
| *A. phagocytophilum* | 2009 | 3 | 125 | 2.4 | 8 | 54 | 14.8 | 8 | 60 | 13.3 |
|  | 2010 | 12 | 386 | 3.1 | 13 | 80 | 16.3 | 9 | 79 | 11.4 |
|  | 2011^*^ | 53 | 668 | 7.9 | 3 | 29 | 10.3 | 2 | 24 | 8.3 |
|  | 2012 | 18 | 417 | 4.3 | 3 | 51 | 5.9 | 9 | 55 | 16.4 |
|  | 2013^*^ | 5 | 201 | 2.5 | 4 | 25 | 16.0 | 1 | 21 | 4.8 |
|  | 2014 | 13 | 289 | 4.5 | 3 | 33 | 9.1 | 5 | 22 | 22.7 |
|  | 2015 | 11 | 355 | 3.1 | 9 | 31 | 29.0 | 8 | 39 | 20.5 |
|  | 2016 | 12 | 381 | 3.1 | 5 | 45 | 11.1 | 4 | 40 | 10.0 |
|  | sum | 127 | 2822 | 4.5 | 48 | 348 | 13.8 | 46 | 340 | 13.5 |

^*^Contains more processed data and a few typos corrected relative to (*28*) and (*24*). Therefore exact numbers are a bit different.

**Additional file 1: Table S3.** The number of cases and incidence (inc) of three tick-borne diseases, Lyme borreliosis, anaplasmosis in sheep, anaplasmosis and babesiosis in cattle, from areas ‘west’ (Sogn & Fjordane) and ‘east” (Akershus and Østfold) in Norway. Incidence is reported per 100000 inhabitant, per 10000 registered ewes and per 10000 cattle based on population sizes given in last columns. CattleOut denote those cattle that are grazing on the outfields, and incOut is the incidence of anaplasmosis and babesiosis in cattle restricted to those cattle grazing outfield.

|  | Lyme | | Anaplasmosis sheep | | Anaplasmosis cattle | | | Babesiosis cattle | | | Population | | | |
| --- | --- | --- | --- | --- | --- | --- | --- | --- | --- | --- | --- | --- | --- | --- |
|  | case | inc | case | inc | case | incOut | inc | case | incOut | inc | Humans | Sheep | CattleOut | Cattle tot |
| A. ‘East’ | | | | |  |  |  |  |  |  |  |  |  |  |
| 2006 | 9 | 1.49 | 0 |  | 0 |  |  | 0 | 0 | 0 | 604102 | 3775 | 4279 | 44316 |
| 2007 | 9 | 1.47 | 0 |  | 0 |  |  | 0 | 0 | 0 | 612558 | 4086 | 4428 | 44141 |
| 2008 | 18 | 2.89 | 0 |  | 0 |  |  | 2 | 4.90 | 0.44 | 622959 | 3974 | 4081 | 45318 |
| 2009 | 11 | 1.74 | 0 |  | 0 |  |  | 1 | 2.26 | 0.22 | 632753 | 4421 | 4429 | 45370 |
| 2010 | 7 | 1.09 | 0 |  | 0 |  |  | 0 | 0 | 0 | 642325 | 3960 | 4679 | 44886 |
| 2011 | 11 | 1.69 | 0 |  | 0 |  |  | 0 | 0 | 0 | 652407 | 3715 | 4868 | 45541 |
| 2012 | 5 | 0.75 | 0 |  | 0 |  |  | 0 | 0 | 0 | 663670 | 3875 | 5346 | 46247 |
| 2013 | 8 | 1.19 | 0 |  | 0 |  |  | 0 | 0 | 0 | 674304 | 4380 | 5249 | 45678 |
| 2014 | 11 | 1.61 | 0 |  | 0 |  |  | 0 | 0 | 0 | 683793 | 5089 | 5746 | 45944 |
| 2015 | 7 | 1.01 | 0 |  | 0 |  |  | 0 | 0 | 0 | 691841 | 5966 | 6528 | 46168 |
| sum | 96 | 1.49 | 0 |  | 0 |  |  | 3 | 0.72 | 0.07 | 648071 | 4324 | 4963 | 45361 |
| B. ‘West’ | | | |  |  |  |  |  |  |  |  |  |  |  |
| 2006 | 21 | 19.69 | 16 | 5.96 | 25 | 10.07 | 4.77 | 33 | 13.29 | 6.30 | 106650 | 26839 | 24833 | 52411 |
| 2007 | 19 | 17.89 | 14 | 5.38 | 17 | 7.12 | 3.34 | 33 | 13.81 | 6.48 | 106194 | 26036 | 23889 | 50933 |
| 2008 | 16 | 15.06 | 29 | 11.54 | 18 | 7.88 | 3.65 | 17 | 7.44 | 3.45 | 106259 | 25128 | 22857 | 49329 |
| 2009 | 20 | 18.79 | 17 | 6.38 | 7 | 3.05 | 1.44 | 36 | 15.67 | 7.41 | 106457 | 26634 | 22970 | 48604 |
| 2010 | 10 | 9.34 | 8 | 3.22 | 18 | 7.81 | 3.76 | 19 | 8.24 | 3.97 | 107080 | 24827 | 23051 | 47832 |
| 2011 | 14 | 12.99 | 10 | 4.17 | 9 | 4.11 | 1.91 | 20 | 9.13 | 4.25 | 107742 | 23972 | 21903 | 47088 |
| 2012 | 8 | 7.39 | 23 | 9.88 | 12 | 5.44 | 2.62 | 21 | 9.52 | 4.58 | 108201 | 23289 | 22065 | 45868 |
| 2013 | 11 | 10.12 | 22 | 9.55 | 9 | 4.03 | 1.99 | 8 | 3.59 | 1.77 | 108700 | 23032 | 22316 | 45178 |
| 2014 | 7 | 6.42 | 20 | 8.88 | 8 | 3.71 | 1.83 | 7 | 3.24 | 1.60 | 108965 | 22526 | 21592 | 43803 |
| 2015 | 7 | 6.41 | 35 | 14.00 | 11 | 5.12 | 2.48 | 20 | 9.31 | 4.51 | 109170 | 24996 | 21489 | 44311 |
| sum | 133 | 12.41 | 194 | 7.90 | 134 | 5.83 | 2.78 | 214 | 9.32 | 4.43 | 107542 | 24728 | 22697 | 47536 |

**Additional file 1: Table S4.** Results from model selection with AIC for the analysis of variation in incidence of tick-borne diseases with negative binomial mixed models for east and west of Norway. A) Lyme borreliosis in humans, B) Babesiosis in cattle, C) Anaplasmosis in cattle, D) Anaplasmosis in sheep. Municipality ID was included as a random term to account for sampling design.

| A. Lyme borreliosis | | | | | | |  | |
| --- | --- | --- | --- | --- | --- | --- | --- | --- |
| Distance to coast | Region | Spatial deer density | Prop. area > 200 m a.s.l. | Prop. area agricultural field | Prop. area human settlement | Region* Prop. area > 200 | AIC | ΔAIC |
| x |  |  |  |  |  |  | 273.6 | 35.7 |
| x | x |  |  |  |  |  | 254.1 | 16.3 |
| x | x | x |  |  |  |  | 254.0 | 16.2 |
| x | x |  | x |  |  |  | 248.6 | 10.7 |
| x | x |  |  | x |  |  | 255.2 | 17.4 |
| x | x |  |  |  | x |  | 248.6 | 10.7 |
| x | x |  | x |  | x |  | 243.3 | 5.5 |
| x | x |  | x |  | x | x | 237.9 | 0.0 |
| B. Babesiosis cattle | | | |  |  |  |  |  |
| Distance to coast | Prop. area > 200 m a.s.l. | Spatial deer density | Prop. area agricultural field | Prop. area human settlement | Density of outfield grazing sheep | Health records | AIC | ΔAIC |
|  |  |  |  |  |  |  |  |  |
|  |  |  |  |  |  |  | 140.1 | 6.5 |
| x |  |  |  |  |  |  | 140.6 | 7.1 |
| x | x |  |  |  |  |  | 142.6 | 9.1 |
| x |  | x |  |  |  |  | 133.6 | 0 |
|  |  | x |  |  |  |  | 135.4 | 1.9 |
| x |  | x |  | x |  |  | 135.5 | 2.0 |
| x |  | x | x |  |  |  | 135.4 | 1.9 |
| C. Anaplasmosis cattle | | | |  |  |  |  |  |
|  |  |  |  |  |  |  | 129.3 | 4.9 |
| x |  |  |  |  |  |  | 129.1 | 4.7 |
| x | x |  |  |  |  |  | 130.7 | 6.3 |
| x |  | x |  |  |  |  | 131.1 | 6.7 |
| x |  |  | x |  |  |  | 124.7 | 0.0 |
|  |  |  | x |  |  |  | 124.4 | 0.3 |
| x |  |  |  | x |  |  | 128.7 | 4.3 |
| x |  | x | x |  |  |  | 125.0 | 0.5 |
| x |  |  | x |  | x |  | 126.7 | 2.3 |
| D. Anaplasmosis sheep | | | | |  |  |  |  |
|  |  |  |  |  |  |  | 135.2 | 21.0 |
| x |  |  |  |  |  |  | 129.0 | 14.7 |
| x | x |  |  |  |  |  | 130.2 | 15.9 |
| x |  | x |  |  |  |  | 127.4 | 13.1 |
| x |  |  | x |  |  |  | 125.2 | 10.9 |
| x |  |  |  | x |  |  | 272.5 | 158.2 |
| x |  |  |  |  | x |  | 116.3 | 2.0 |
| x |  |  |  |  | x | x | 114.3 | 0.0 |
| x |  | x |  |  | x | x | 115.5 | 1.2 |

**
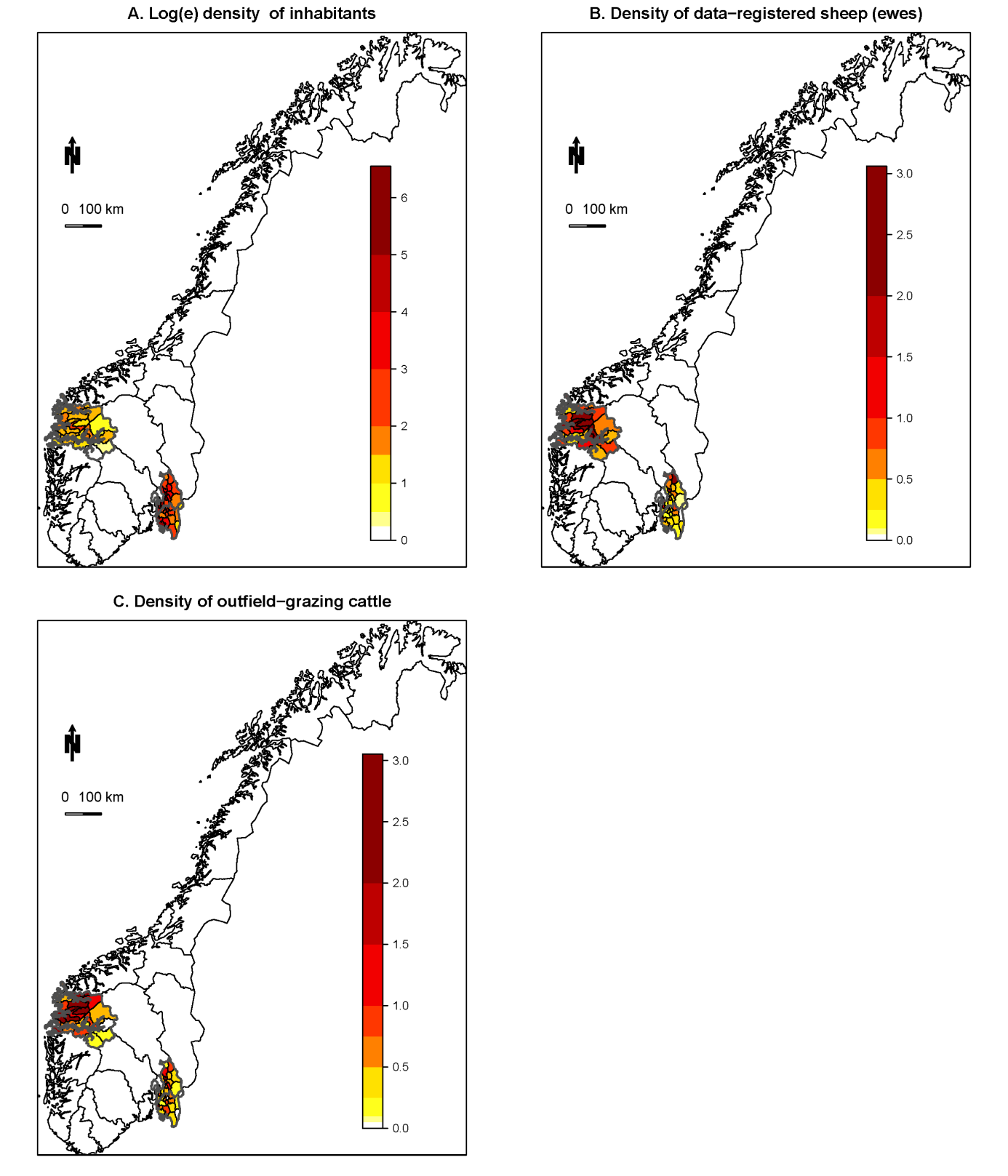
Additional file 1: Fig. S1.** The population density of humans, sheep and cattle in the western and eastern region of Norway.

**
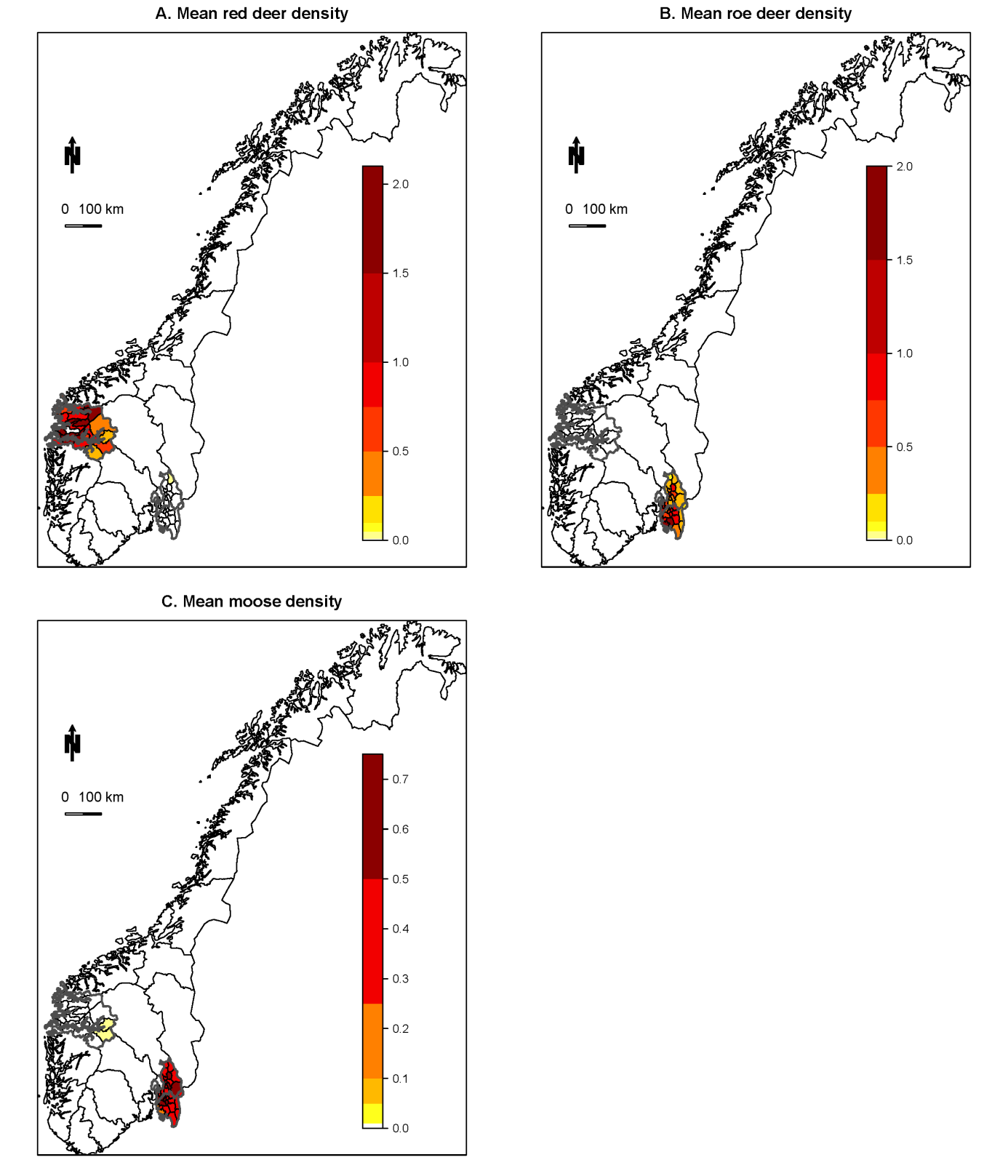
Additional file 1: Fig. S2.** The population density of red deer, moose, and roe deer in the western and eastern region of Norway.
